# Chronic pericarditis and recurrent pericardial effusion of unknown origin in a kitten: a case report

**DOI:** 10.3389/fvets.2024.1347083

**Published:** 2024-06-17

**Authors:** Nicole Weingand, Chang He, Llorenç Grau-Roma, Katja-Nicole Adamik

**Affiliations:** ^1^Division of Small Animal Emergency and Critical Care, Department of Clinical Veterinary Science, Vetsuisse Faculty, University of Bern, Bern, Switzerland; ^2^Institute of Animal Pathology, Vetsuisse Faculty, University of Bern, Bern, Switzerland

**Keywords:** pericardial effusion, pericarditis, cardiopulmonary arrest, feline, cardiopulmonary resuscitation

## Abstract

A 3-month-old kitten was presented after successful cardiopulmonary resuscitation, including a presumed intracardial injection by its primary care veterinarian. Throughout the subsequent hospitalization in the intensive care unit, the cat exhibited recurrent hemorrhagic pericardial effusions, along with concurrent pleural and abdominal effusions, resulting in multiple clinical deteriorations, necessitating pericardiocentesis and thoracocentesis. Despite more than 3 days of intensive care, the cat experienced another cardiopulmonary arrest with unsuccessful attempts to achieve the return of spontaneous circulation. Necropsy and histopathological findings revealed diffuse chronic-active pericarditis and hemorrhagic pericardial effusion, a condition that has not been documented in the context of cardiopulmonary resuscitation or pericardiocentesis.

## Introduction

Pericarditis and pericardial effusion (PE) are rare conditions in cats (1). As potential causes for PE, feline infectious peritonitis (FIP) (2, 3), congestive heart failure (4), neoplasms such as lymphoma (5) or sarcoma (6), and inflammatory diseases (7) are described. Regardless of this, cardiopulmonary resuscitation (CPR) and especially intracardiac injections (ICIs) are known risk factors for iatrogenic damage to the heart and pericardium in dogs, potentially leading to PE (8). Pericarditis can be linked to conditions such as bacterial infections (9–11), FIP (2), migrating foreign bodies (12, 13), hypereosinophilic syndrome (14), lymphoma (15), and peritoneopericardial diaphragmatic hernia repair (16). Pericarditis can be effusive or constrictive in nature; however, a combined form, effusive-constrictive pericarditis (ECP), has been described in human and veterinary medicine (17–19). ECP has been reported as an uncommon cause of pericarditis in humans, with a prevalence of 1.3 to 14.8% (20). The most common etiologies in humans are idiopathic, post-radiation, tuberculous pericarditis, and neoplastic diseases (21). In veterinary medicine, reports of ECP are rare. The first case report, published in 1996, describes a dog with an effusive-constrictive pericardial disease secondary to an osseous metaplasia of the pericardium (18). Heinritz et al. (19) published a case series of 17 dogs with ECP, secondary to *Coccidioides immitis* infection, and Woolley et al. (22) published a case report of a 7-year-old Jack Russel Cross with a 5-week history of ascites and the diagnosis of ECP just the year after.

The following case report portrays a 3-month-old kitten with recurrent cardiac tamponade and pericarditis. To the best of our knowledge, this is the first report of a cat with clinical and pathological findings suggestive of constrictive pericarditis with recurrent PE.

## Case presentation

A 3-month-old, 1.6 kg, female intact Domestic Shorthair cat was referred to the small animal clinic, Vetsuisse faculty, University of Bern, after successful cardiopulmonary resuscitation (CPR) by a referring veterinarian. The cat was adopted from a shelter 1 week before the presentation and has experienced diarrhea since its adoption. The cat was referred to the consulting veterinarian due to a worsening condition of lethargy and persistent diarrhea. During the presentation at the referring veterinarian, the cat was in lateral recumbency, tachypneic, and severely hypothermic with a non-measurable body temperature. Initial biochemistry analyses revealed hyperkalemia, hyponatremia, hypochloremia, increased urea and hyperphosphatemia, and increased alanine aminotransferase (Table 1a). Plasma albumin, alkaline phosphatase, glucose, gamma-glutamyl transferase, bilirubin, amylase, and lipase, as well as hematology parameters, were within normal limits. Approximately 15 min after starting an intravenous (IV) constant rate infusion with normal saline (rate 5 mL/kg/h) and glucose supplementation, the cat experienced a cardiopulmonary arrest (CPA). CPR utilizing a single intracardiac injection (ICI) of low-dose epinephrine (0.01 mg/kg), followed by an additional administration via the IV route, along with 8 min of chest compressions and supplemental oxygen, was successful. After progressive warming and stabilization, the cat was referred for further management to the small animal clinic of a veterinary teaching hospital.

**Table 1:** Venous blood gas analysis and biochemistry results: (1a) at the primary care veterinarian, (1b) on admission, (1c) at the time of first clinical deterioration on the morning of day 2, (1d) prior to evacuation of pericardial effusion on day 2; AG, anion gap; ALAT, alanine aminotransferase.

Parameter	Values (1a)	Values (1b)	Values (1c)	Value (1d)	Reference range
pH		7.335	7.350	7.310	7.380–7.400
pvCO_2_		25.3	32.4	43.0	36.0–39.0 mmHg
pvO_2_		46.9	29.3	38.1	33.0–37.0 mmHg
HCO_3_^−^		13.2	17.4	21.2	21–24 mmol/L
BE		−11.2	−7.4	−4.8	−3 - +3
Na^+^	136	133	134	141	148–156 mmol/L
K^+^	6.70	4.53	5.03	3.63	3.4–4.6 mmol/L
Ca^++^		1.00	1.1	1.1	1.1–1.3 mmol/L
Cl^−^	98	107	108	105	115–125 mmol/L
AG		16.8	13.5	18.9	16–20
Lactate		2.26	2.0	5.98	< 2.0 mmol/L
Phosphorus	160				45–104 mg/L
Urea	1.79				0.34–0.70 g/L
ALAT	225				12–115 U/L
Creatinine		67			53–141 μmol/L

The cat was brought in on a weekend, limiting the availability of full diagnostic resources. Upon arrival at our institution, the patient was in lateral recumbency, severely lethargic but reactive to pain, and severely tachycardic (heart rate = 300 bpm) with poor pulse quality and hyperthermic [rectal temperature, 40°C (104°F)]. The respiratory rate was mildly increased (respiratory rate = 44 bpm) with normal respiratory effort, and mucous membranes were slightly tacky and pale pink with a capillary refill time of 1.5 s. Skin turgor was mildly reduced. Auscultation of the heart and lungs revealed slightly reduced lung sounds and normal heart sounds.

The point of care ultrasound (POCUS) of the heart and lungs revealed a mild hypoechoic PE with small hyperechoic particles, without pericardial tamponade, defined as the absence of signs of right atrial collapse in diastole or early systole, and a moderate amount of anechoic pleural effusion. The abdomen was soft and not painful, and there was a scant amount of anechoic-free peritoneal fluid in the abdominal POCUS. The urinary bladder was small.

The venous blood gas analysis revealed a moderate decrease in HCO_3_^−^ with a mildly decreased pH. Further findings on initial bloodwork included normokalemia, hyponatremia, hypochloremia, mild hyperlactatemia, and a normal anion gap. Plasma creatinine concentration was within normal limits (Table 1b).

Due to the small amount of PE, the lack of cardiac tamponade, and the small patient size, it was decided against performing a diagnostic pericardiocentesis. The tentative diagnosis at this time was hypovolemia due to diarrhea leading to decompensated hypovolemic shock with subsequent CPA. Furthermore, the tri-cavitary effusion was suspected to be due to CPR and possible pericardial hemorrhage after ICI at the referring veterinarian. Initial treatment consisted of supplemental oxygen (flow rate of 2 L/min), a fluid bolus of 5 mL/kg of normal saline, followed by IV normal saline at a rate of 2 mL/kg/h for fluid maintenance. During stabilization, the cat’s heart rate decreased to 200 bpm, and the temperature normalized to 38.6°C (101.5°F). To prevent hyperkalemia, a constant rate infusion of a specially prepared solution was administered over 36 h. This solution, compounded in a 50-ml syringe, comprised the following: 25 mL of sodium bicarbonate 8.4% (Natrium Bicarbonate Braun 8.4%®; B. Braun Medical AG; CH-6204 Sempach, Switzerland), 12.5 mL of dextrose 50% (Glucose 50%; B. Braun Medical AG; CH-6020 Emmenbruecke, Switzerland), and 12.5 mL of sterile water (Aqua ad injectabilia; Dr. Bichsel AG; CH-3800 Interlaken, Switzerland), resulting in a solution with 1 mmoL/mL NaHCO3 and 1.5 g/mL glucose, administered at a rate of 0.25 mL/kg/h. For prophylaxis of nausea, the cat also received Ondansetron 0.3 mL/kg IV (Ondansetron, Labatec Pharma SA, CH-1217 Meyrin). The cat was placed in an oxygen cage with a 40% fraction of inspired oxygen.

In the subsequent hours, the cat’s overall condition markedly improved. It was alert and responsive, with vital signs and Doppler-measured systolic blood pressure within normal limits. The cat urinated as expected, showed no signs of defecation, and demonstrated a robust appetite. The patient remained clinically stable for the next 24 h, still showing a moderate pericardial and pleural effusion but without evident signs of respiratory or cardiovascular compromise. Fluid therapy was regularly adjusted according to electrolyte changes.

**Figure 1:**
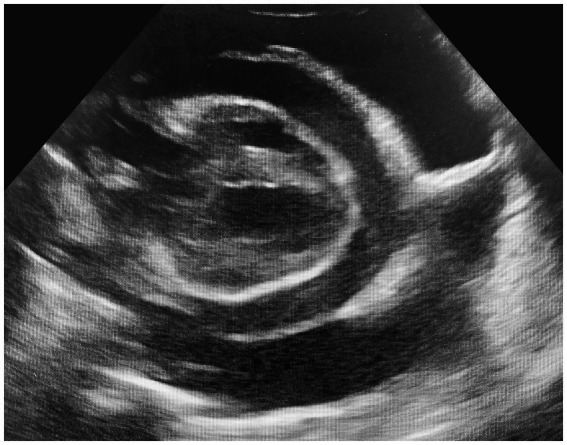
Moderate amount of pericardial and pleural effusion prior to therapeutic pericardiocentesis and thoracocentesis.

Approximately 38 h after admission, the cat exhibited mild signs of respiratory distress, characterized by a normal respiratory rate but a notable increase in respiratory effort. Repeated blood gas analysis revealed a resolution of the previous metabolic acidosis, mild hypocapnia, normalization of HCO_3_^−^, and relatively unchanged electrolyte imbalances (Table 1c). Thoracic POCUS revealed a moderate amount of pleural effusion. To address this, a thoracocentesis was conducted under sedation (using butorphanol at a dose of 0.2 mg/kg IV), yielding 30 mL of modified transudate. Subsequently, the cat’s overall condition and respiration promptly improved, returning to a normal respiratory rate and effort. Three hours later, the cat experienced a sudden episode of lateral recumbency, was minimally responsive, and showed severe hypothermia (32.6°C). Repetition of blood gas analysis revealed mild metabolic acidosis, moderate hyperlactatemia, mild hyponatremia, and hypochloremia but was otherwise within normal limits (Table 1d). The POCUS revealed moderate pericardial, significant pleural, and mild abdominal effusion (Figure 1). Due to the patient’s clinical deterioration, pericardiocentesis (6 mL), thoracocentesis (75 mL), and diagnostic abdominocentesis were performed.

Fluid analysis of the PE indicated a recent hemorrhage (PCV 29%), while the pleural and abdominal effusion was consistent with a modified transudate (cell-poor, lucent, specific gravity 1.018, total solids of 30 g/L), with a cell differential count of 58% non-degenerate neutrophils, 4% small lymphocytes, 38% macrophages, rare reactive mesothelial cells were also observed, and no micro-organisms were found. After the removal of the pericardial and pleural effusion, the cat’s cardiovascular condition showed immediate improvement. To prevent possible hyperfibrinolysis and hemorrhage, tranexamic acid (at a dosage of 10 mg/kg over 20 min IV) was administered. The cat was cardiovascularly stable (heart rate = 180 bpm, respiratory rate = 32 bpm, mucous membranes pink and moist, and Doppler blood pressure was 108 mmHg) for the next 8 h, with slight pleural and pericardial effusion on the POCUS.

The following day (day 3), the cat had a thorough clinical examination, and the heart, thorax, and abdomen were additionally re-examined using the POCUS, revealing a mild-to-moderate pericardial and pleural effusion as well as mild ascites. There were no signs of cardiac tamponade at this time. Due to the good clinical condition of the cat, no further thoraco- or pericardiocentesis was performed. A repeated CBC and biochemistry analysis was unremarkable, except for persistent mild hyponatremia, hypochloremia, and mild hypoalbuminemia [26.6 g/L (30–42)]. A fecal sample was sent for parasitological testing and revealed infection with *Cystoisospora ohioensis-burrowsi*, which was treated with Toltrazuril (at a dose of 10 mg/kg PO).

Twelve hours later, the cat experienced acute respiratory distress. On clinical examination, lung sounds were muffled, and the POCUS revealed a massive pleural effusion. A total of 70 mL of modified transudate was subsequently evacuated via thoracocentesis. The pericardium was mild-to-moderately filled, and no pericardiocentesis was performed. Another 2 h later, the cat was in lateral recumbency with muffled heart sounds, agonal breathing pattern, and bradycardia. Immediate endotracheal intubation and pericardiocentesis were performed, but the cat experienced CPA. Cardiopulmonary resuscitation efforts, consisting of chest compressions, manual ventilation, and administration of epinephrine (0.01 mg/kg IV) and atropine (0.05 mg/kg IV), led to a short occurrence of ventricular pulseless tachycardia. However, during the preparation of the patient (clipping) and setup of the defibrillator, the rhythm changed to bradyarrhythmia and ultimately asystole. A return of spontaneous circulation (ROSC) could not be achieved.

**Figure 2:**
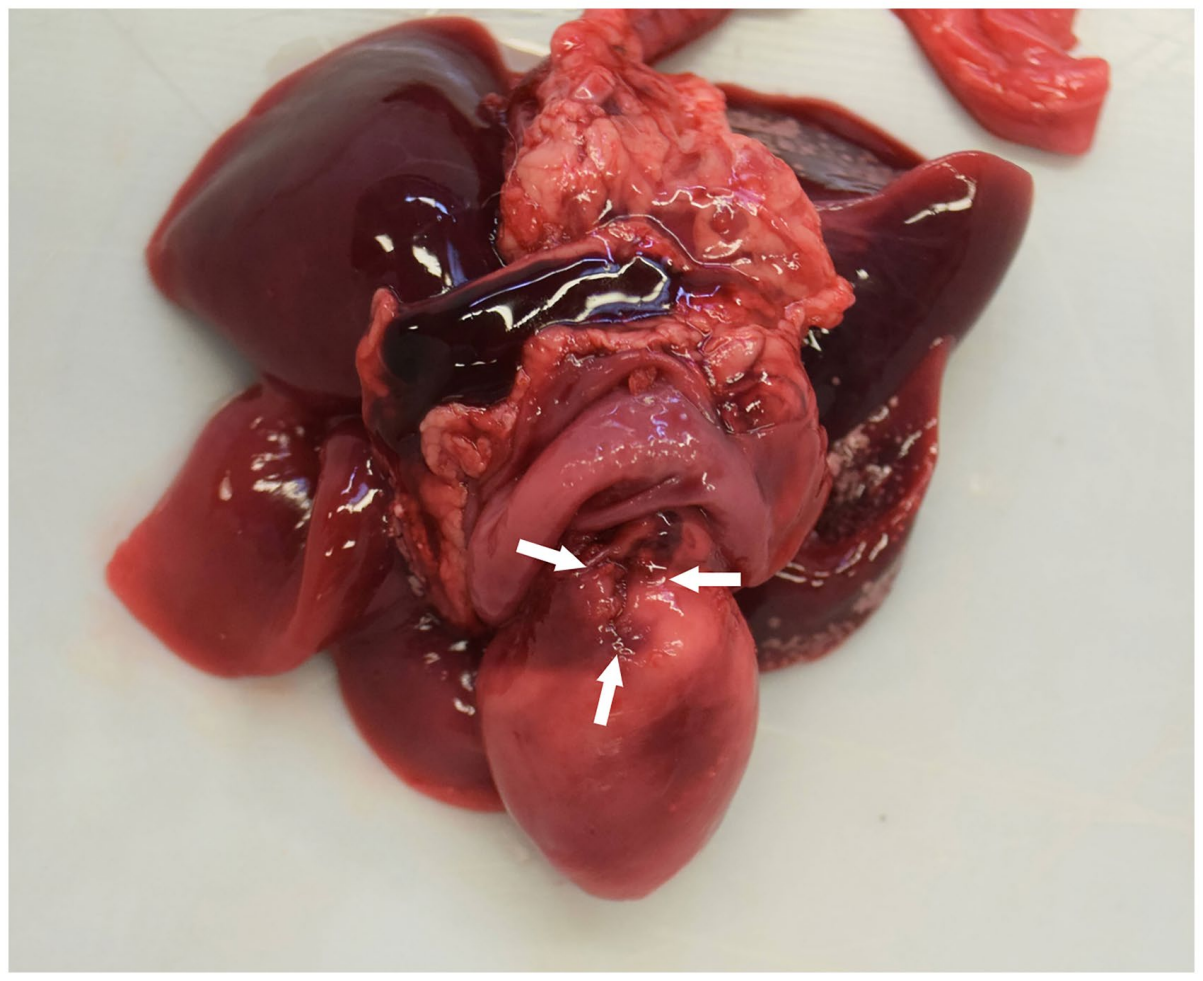
Heart and lungs. The visceral and the parietal pericardium are diffusely and moderately thick with a rough surface. An irregularly shaped notch of 3 × 10 mm is visible on the epicardium (arrows).

**Figure 3:**
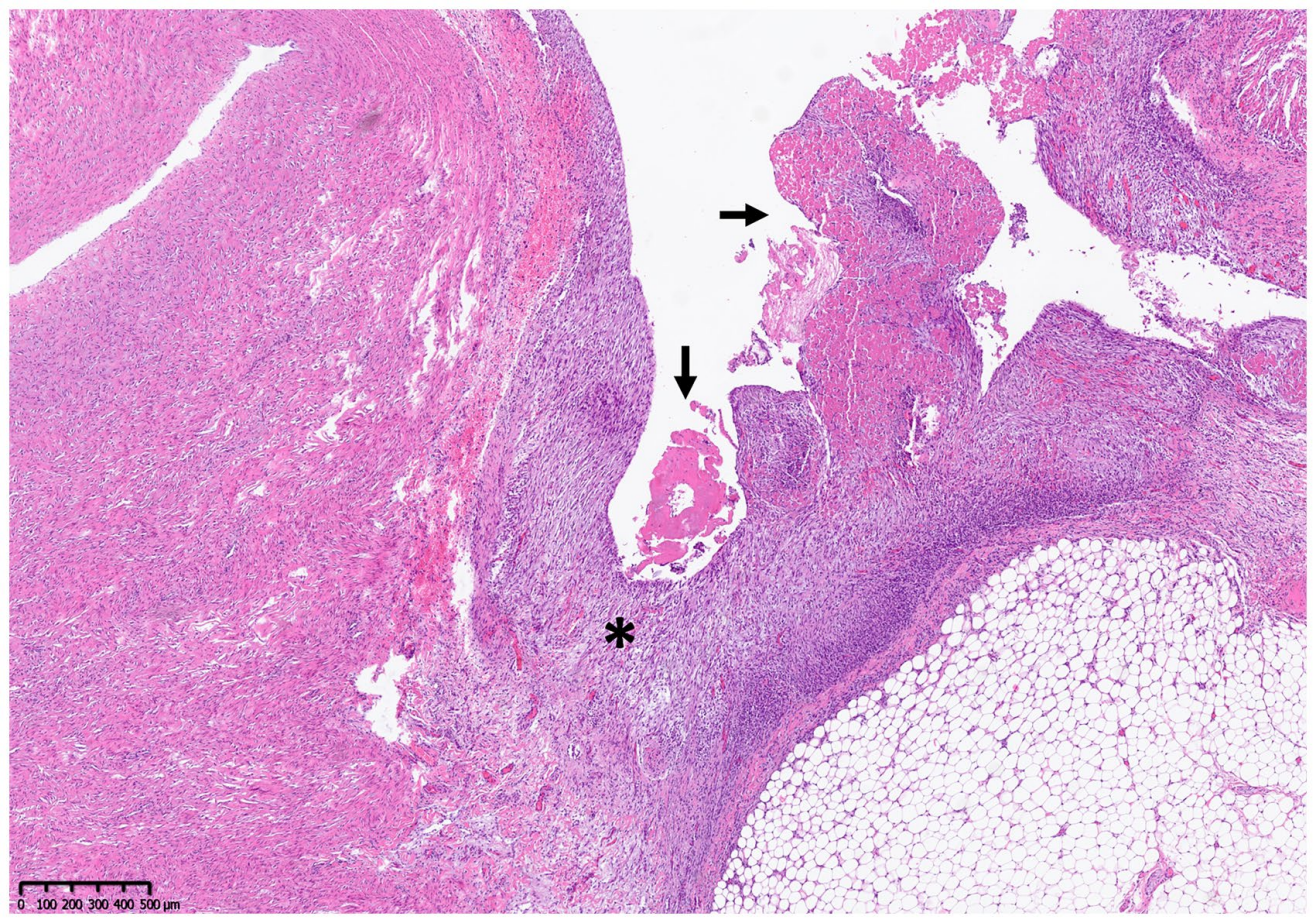
Histology of the myocardium and pericardium. The visceral pericardium is diffusely and markedly thickened by prominent granulation tissue formation (asterisk), and multifocal aggregates of fibrin are seen on its surface (arrow).

A full post-mortem examination was performed within 48 h after death. Gross examination revealed pleural (20 mL), pericardial (2 mL), and abdominal (15 mL) effusion. The pleural and abdominal effusions were mildly yellowish and serosanguinous in character; in the pericardium, a hemorrhagic effusion was found. The pericardium was diffusely thickened and showed a rough appearance. A focal irregularly shaped notch of 3 × 10 mm was identified in the epicardium and the myocardial surface, which was interpreted as a focal rupture (probably the ICI) (Figure 2). In addition, mild-to-moderate subcutaneous edema on the ventral abdomen and moderate alveolar pulmonary edema were present. The histological examination revealed diffuse chronic-active pericarditis with prominent granulation tissue formation, infiltration of some neutrophils, fibrin exudation, and small hemorrhages (Figure 3). The liver showed mild-to-moderate acute, multifocal, mostly paracentral, hepatocellular necrosis. No other relevant findings were observed. To investigate the possible involvement of FIP as etiology, immunohistochemistry for feline coronavirus (FCoV) was performed on the tissues of the heart, brain, and liver, which gave negative results.

## Discussion

A 3-month-old kitten was presented to a veterinary teaching hospital after successful CPR at the primary care veterinarian. The cat initially showed a good response to post-cardiac arrest care. The exact cause for CPA could not be definitively determined. The initial suspicion was hypovolemic shock due to a 1-week history of diarrhea, which then led to hypovolemia, acute circulatory failure, and CPA. Pericardial hemorrhage was suspected to be the consequence of ICI of epinephrine during CPR at the primary care vet, leading to subsequent pleural and abdominal effusion. Notably, the cat displayed only mild signs of hypovolemia and dehydration upon arrival at our institution. However, the volume of IV fluid therapy administered by the primary care vet was unknown and could therefore bias our interpretation of the patient’s volume and hydration status. During hospitalization (3.5 days), the cat experienced a total of three episodes of clinical deterioration with respiratory distress, signs of cardiovascular impairment, and recurrences of pleural and pericardial effusions, ultimately leading to another CPA and a failure to achieve ROSC. Pericardiocentesis was performed twice and presented a hemorrhagic effusion with a hematocrit of 29%. Due to the uncommon clinical course of this patient, a necropsy and histopathological examination for further investigation were performed. This examination revealed severe pericarditis with signs of inflammation and hemorrhage in combination with a rough epicardial surface. Thus, our initial hypothesis of solely pericardial hemorrhage resulting from intracardiac injection was not entirely corroborated by the histopathological findings.

Pericarditis and cardiac tamponade, in general, are rare conditions in cats. Conditions leading to PE and/or pericarditis include cardiomyopathies, neoplasia, infectious or inflammatory causes, as well as migrating foreign bodies and trauma (23). Cardiomyopathies, neoplasia, and foreign bodies have been excluded as potential causes in this case based on the findings from macroscopic and microscopic post-mortem examinations. The main infectious cause of pericarditis in cats is due to FIP, which was ruled out by immunohistochemistry of several tissue samples. Other infectious conditions, such as bacteriemia or sepsis, could lead to pericarditis, which is considered unlikely in this case. Although a blood culture was not performed, clinical (normotension, normothermia, normocardia, normopnoea, and pink mucous membranes), laboratory (normal leukocyte count without left shift, low serum amyloid A, and normal bilirubin concentrations), and post-mortem findings in this kitten would not fit a septic patient. Trauma as a potential cause cannot be ruled out due to the fact that the cat received CPR with ICI and pericardiocentesis. Since post-mortem examination revealed signs of chronic pericarditis, it would be a possibility that the cat suffered from a chronic pericarditis of unknown clinical significance at the time of presentation to the referring veterinarian and that the ICI led to a recurrent hemorrhage with clinical deteriorations and cardiac tamponades in the days after CPR.

Drawing from human literature, a clinical condition is described with cardiac tamponade in conjunction with constriction of the heart by pericarditis, termed *effusive constrictive pericarditis* (ECP). In two case series of human patients, the main clinical symptoms of ECP were dyspnea, left-sided chest pain, ascites, hepatomegaly, and ankle edema. The diagnosis was made by cardiac catheterization and measurements of the right atrial pressure (RAP) before and after pericardiocentesis. A temporary clinical improvement could be achieved via pericardiocentesis; however, RAP remained abnormal in patients with ECP because the fluid accumulation is only partially responsible for the increased pressure and clinical presentation. Long-term improvement was achieved by cardiac surgery. During surgery, restriction of the ventricular relaxation during the diastolic phase could be seen, which improved after epicardiectomy (17, 24). In a large prospective study, the prevalence of ECP among all patients with pericardial disease was only 1.3%, whereas in patients with cardiac tamponade, the prevalence rose to 6.9%. The low prevalence was primarily explained by ECP being a rare condition, and secondarily by the difficulty of diagnosing this condition (20). A measurement of the RAP and the pressure inside the pericardial sac must be obtained before and after pericardiocentesis to confirm that the intrapericardial pressure remains increased even after drainage of the pericardial effusion (25, 26). Zagol et al. (27) described typical echocardiographic and cardiac-MRI changes in people with ECP based on a case report. These findings are based on the authors’ experience and have not been established by a prospective study and should therefore be interpreted cautiously.

The etiology of ECP in humans is multifaceted, encompassing various factors, including idiopathic and inflammatory causes, as well as radiation therapy (26, 28). The possibility that our patient developed an ECP as a result of an infectious or inflammatory origin cannot be completely ruled out because blood cultures or other bacteriological tests were not performed; however, neither the repeated clinical examinations nor the CBC and SAA results support this theory in our case. The cat was clinically stable, bright, and alert between deteriorations, which did not raise the suspicion of an ongoing systemic inflammatory or infectious disease process. In addition, post-mortem immunohistochemical testing for FCoV-antigen performed from various tissues was negative. Due to the fact that no disease process could be identified as a potential trigger, an idiopathic ECP must be considered; however, idiopathic pericarditis or iatrogenic pericarditis due to the ICI could also be an explanation. A study including 205 patients receiving echocardiography before and after pericardiocentesis found that in patients with ECP, cardiac tamponade was more frequently observed; however, the volume obtained from pericardiocentesis was significantly less than in patients without constriction (29). In general, clinically relevant PE and cardiac tamponade in cats are rare. Pericardial effusions are usually associated with small volumes and do not cause clinical signs of circulatory impairment. A study investigating PE in adult cats found an average effusion volume of 5.9 mL (range 2.0–25 mL) without signs of cardiac impairment (23). The exact amount of fluid necessary to cause cardiac tamponade cannot be determined because it is dependent on fluid volume, elasticity of the pericardium, and the time span of filling. During slow fluid accumulations, patients usually tolerate larger volumes in comparison to fast-occurring effusions (30). In the present case, a pericardial effusion of 6 mL is considered a moderate pericardial effusion, which supports the theory of a constrictive component leading to cardiac tamponade. The development of ascites and pleural effusion, both consistent with modified transudates, aligns well with right-sided cardiac dysfunction. Given that the cat underwent multiple daily examinations for pericardial effusion, which consistently appeared mild-to-moderate, a constrictive component is suspected. While the cat’s clinical picture and the histopathological results of diffuse chronic-active pericarditis with prominent granulation tissue formation support our suspicion of a constrictive component in this case of pericarditis, the effusive aspect remains difficult to interpret. Recurrent hemorrhage after ICI would be a likely explanation, especially as the effusion was always hemorrhagic. For a definitive diagnosis of ECP, measuring the RAP and intrapericardial pressure both before and after evacuating the pericardial effusion would be required. Both measurements are not routinely performed in small animal medicine and are invasive. Additionally, due to the out-of-hour presentation of the cat, a board-certified cardiologist was not available for a complete echocardiography. However, POCUS was performed by a trained veterinarian under the supervision of a board-certified emergency and critical care (ECC) specialist. Measure of central venous pressure (CVP) was not considered due to patient size and initial clinical improvement. The appearance of the jugular veins at times of clinical deterioration was unfortunately not documented. Therefore, an increased RAP cannot be confirmed and remains a suspicion. As neither of the aforementioned measurements were performed, the diagnosis of an ECP in that cat cannot be made. Unfortunately, the lack of documentation of ante-mortem findings and lack of further diagnostics limit the possibility of reaching a definitive diagnosis in this case. The interpretation of the histopathological results, especially in light of the short clinical history, is also difficult. It is unlikely that the cat developed signs of chronic-active pericarditis due to CPR efforts and ICI within only 3 days. Therefore, a pericarditis of unknown origin must have been present before the initial CPA. However, the question arises as to what the original trigger for this pericarditis was and why the cat was apparently healthy before the development of gastrointestinal symptoms and later lethargy. Additionally, it would be interesting, if the cat had already presented with PE at the primary care veterinarian, to further narrow down the reasons for PE. If PE had been present before CPR, an effusive component would be more likely. However, if not, the recurrent hemorrhagic PE may be linked to the ICI during initial CPR. Furthermore, resuscitation efforts can cause iatrogenic damage to the patient’s body. In 2021, a study investigated iatrogenic lesions after CPR in dogs and found an overall occurrence of hemopericardia in 4.8% of patients. Notably, all 8 patients who received ICI exhibited complications, including hemopericardium, hemothorax, hemomediastinum, and cardiac and pulmonary hemorrhage (8). In the cat reported here, both scenarios could be possible. An iatrogenic damage leading to recurrent pericardial hemorrhage cannot be ruled out. However, it is essential to consider that the cat’s initial CPA may have resulted from a pre-existing pericardial effusion and tamponade, possibly related to an ECP, and the ICI was relieving the pressure from the pericardium, like in a pericardiocentesis. Consequently, the initial ICI might have led to ROSC in the first place through the emptying of the pericardial effusion, the resulting reduction in RAP, and the consequent improvement in cardiac filling and cardiac output.

## Conclusion

A 3-month-old kitten presented to a veterinary teaching hospital after successful CPR, including ICI. The patient exhibited recurrent hemorrhagic pericardial effusions as well as signs of right-sided cardiac impairment, with cardiovascular stable phases in between pericardiocentesis. Although under close clinical observation and regular POCUS rechecks, the patient experienced another rapid clinical deterioration, leading ultimately to another CPA and death despite CPR efforts. The clinical presentation, together with pathological findings, are suggestive of constrictive pericarditis with recurrent PE. Whether these effusions were related to ongoing hemorrhage after ICI or could be due to an ECP remains unknown, which emphasizes the importance of diagnostics such as jugular vein assessment, CVP measurements, echocardiography, and cardiac catheterization in the work-up of recurrent PE in cats.

## Data availability statement

The original contributions presented in the study are included in the article/supplementary material, further inquiries can be directed to the corresponding author.

## Ethics statement

Ethical approval was not required for the studies involving animals in accordance with the local legislation and institutional requirements because it is a case report written post mortem. During hospitalization the cat received evidence-based best practice medical treatment. Written informed consent was obtained from the owners for the participation of their animals in this study.

## Author contributions

NW: Writing – original draft. CH: Writing – review & editing. LG-R: Writing – review & editing. K-NA: Writing – review & editing.
